# Plant Metabolomics: An Indispensable System Biology Tool for Plant Science

**DOI:** 10.3390/ijms17060767

**Published:** 2016-06-01

**Authors:** Jun Hong, Litao Yang, Dabing Zhang, Jianxin Shi

**Affiliations:** 1 Joint International Research Laboratory of Metabolic & Developmental Sciences, Shanghai Jiao Tong University–University of Adelaide Joint Centre for Agriculture and Health, School of Life Sciences and Biotechnology, Shanghai Jiao Tong University, Shanghai 200240, China; 13605151497@163.com (J.H.); yylltt@sjtu.edu.cn (L.Y.); zhangdb@sjtu.edu.cn (D.Z.); 2 Plant Genomics Center, School of Agriculture, Food and Wine, University of Adelaide, Waite Campus, Urrbrae, South Australia 5064, Australia

**Keywords:** primary and secondary metabolism, mQTL, mGWAS, metabolic engineering, crop improvement

## Abstract

As genomes of many plant species have been sequenced, demand for functional genomics has dramatically accelerated the improvement of other omics including metabolomics. Despite a large amount of metabolites still remaining to be identified, metabolomics has contributed significantly not only to the understanding of plant physiology and biology from the view of small chemical molecules that reflect the end point of biological activities, but also in past decades to the attempts to improve plant behavior under both normal and stressed conditions. Hereby, we summarize the current knowledge on the genetic and biochemical mechanisms underlying plant growth, development, and stress responses, focusing further on the contributions of metabolomics to practical applications in crop quality improvement and food safety assessment, as well as plant metabolic engineering. We also highlight the current challenges and future perspectives in this inspiring area, with the aim to stimulate further studies leading to better crop improvement of yield and quality.

## 1. Introduction

Plants produce large numbers of metabolites of diversified structures and abundance that play important roles in plant growth, development, and response to environments. These diverse small molecular weight metabolites, the chemical base of crop yield and quality, are also valuable nutrition and energy sources for human beings and live stocks [1]. Generally, these metabolites are classified into primary and secondary metabolites. The former are indispensable for the growth and development of a plant, while the latter are not essential but are crucial for a plant to survive under stress conditions by maintaining a delicate balance with the environment. In addition, primary metabolites are highly conserved in their structures and abundances while those of secondary metabolites differ widely across plant kingdoms [2]. The diversity of plant metabolites and the likely complicated regulatory mechanism highlight the necessity to explore the underlying biochemical nature [1].

The output of plant metabolomics depends largely on its methodologies and instrumentations to comprehensively identify, quantify, and localize every metabolite. Actually, it is very challenging because of the complexity of the diverse metabolic characteristics and abundances of molecules. Fortunately, albeit the fact that accurate and exhausted analysis of the whole metabolome of a biological sample seems currently impossible, methodologies and instrumentations of plant metabolomics have been developing rapidly [3]. At present large scale analysis of highly complex mixtures are enabled by a series of integrated technologies and methodologies, such as non-destructive NMR (nuclear magnetic resonance spectroscopy), mass spectrometry (MS) based methods including GC–MS (gas chromatography–MS), LC–MS (liquid chromatography–MS) and CE–MS (capillary electrophoresis–MS), and FI-ICR–MS (Fourier transform ion cyclotron resonance–MS) [4,5]. Assisted by other technologies of sampling, metabolomics could be performed in the subcellular level and even in a single cell [6,7,8,9]. These analytical approaches have shown their potential power in plant metabolomic studies in many common plant species including staple food crops such as tomato, rice, wheat, and maize for various purposes [10,11,12,13]. However, because of the intrinsic limitation of each analytical platform, combined approaches are increasingly used in metabolomics analysis.

Although metabolomics is downstream of the other functional genomics (transcriptomics and proteomics), the practical size of the metabolome of a species, unlike transcriptome or proteome, cannot be speculated directly by known genomic information via central dogma. Therefore, metabolomics is used to obtain a large amount of valuable information for the discovery of genes and pathways through accurate and high throughput corollary peak annotation via snapshotting the plant metabolome [14]. It seems that there is a complicated regulatory network among these small molecules in plants, and by detecting the interactions among these metabolites, metabolomic analysis contributes significantly to the understanding of the relation between genotype and metabolic outputs by tackling key network components [15]. Such kinds of metabolomic analysis, integrated with transcriptomic analysis, have been successfully applied to investigate the coordinated rules of metabolic fluxes and metabolite concentrations in plants [15,16]. Recently, high throughput and low-cost approaches have been used to achieve huge omics data output in a short time, and further to reconstruct the metabolic models in microbial organisms [17]. However, integration of sequential multiple omics data to understand plant development remain challenging, since the relationship between each of the omics is complex and not always linear. Nevertheless, plant metabolomics has become a powerful tool to explore various aspects of plant physiology and biology, which broadens significantly our knowledge of the metabolic and molecular regulatory mechanisms regulating plant growth, development and stress responses, and the improvement of crop productivity and quality. In this review, we summarize our current understanding of plant physiology and biology in the context of metabolites and metabolic networks. The important roles of inherent genetic factors governing the natural metabolic variation among plants are highlighted, the application of plant metabolomics in crop improvement, and its future prospective are also discussed.

## 2. Using Plant Metabolic Phenotype to Reveal the Function of Genes in the Plant Genome

With the advance of sequencing technology, dozens of plant species have been sequenced. To comprehensively understand functional genomics regarding plant development, the importance of advanced tools of metabolomics, together with QTL (quantitative trait locus) analysis, GWAS (genome-wide association study), and knock-out/down technology, has been increasingly recognized within the plant science community.

### 2.1. From mQTL to mGWAS: Hunting for Candidate Genes Correlated to Metabolic Phenotype in Genetic Variation

In plants, it is well known that QTLs are distributed in many regions of the chromosome and large numbers of alleles occur in the process of domestication. Molecular breeding benefits from the fragment with preponderant genes that leads to high productivity or quality. Compared with the few participants and unfulfillable crosses-designed in human genetics, plants are more suitable for linkage analysis. However, one of the limitations of complex QTL mapping is the acquirement of precise phenotype data. Although high throughput plant phenotyping platforms and corresponding plant phenomics have offered and integrated a set of novel technologies, more details of complex plant phenotypes still need to be mined. More recently, some specific traits like metabolic variants in large-scale omics data have been taken into analysis in human disease and mouse studies [18,19], and shows more advantages than classic macroscopical phenome in disease and pharmaceutical studies because it provides much more information [20]. Therefore, using the metabolic phenotype to study genetic variation may deepen our understanding of plant biology from a metabolomic viewpoint. Table 1 summarizes currently conducted researches. In *Arabidopsis*, the analysis of 369 recombinant inbred lines and 41 introgression lines indicated that the metabolite heterosis is primarily contributed by epistasis [21]. In tomato, metabolite profiling in seeds of 76 introgression lines in two consecutive harvest seasons revealed the presence of 30 metabolite quantitative trait loci (mQTLs) and dissected partial mechanisms, underlying the variational contents of main primary metabolites [22]. Similar mQTL analyses have been performed in other plant species, such as wheat, rice, and rape [23,24,25], however, genetic bases of the metabolomics diversity in plants remain to be further uncovered. In QTL analysis, the heritability of given phenotype (broad-sense heritability) and the *r*^2^ of the individual locus linked to a given phenotype (the effect size of locus), two important parameters, are usually evaluated. In metabolome-based QTL study, primary metabolites often have high heritability, and the secondary metabolite loci have higher *r*^2^ for a metabolic phenotype than primary metabolites [26].

Besides linkage analysis, QTLs can also be identified through association analysis [52]. Compared with most artificial mapping populations that are constructed by crosses between two parental accessions, association analysis populations are composed of large numbers of natural accessions containing more genetic variants as well as potential for the identification of unknown phenotype-associated loci in the plant genome. Benefiting from the development of next-generation sequencing technologies, metabolome-based GWAS (mGWAS) has been used to understand genetic mechanisms underlying metabolic diversity and their associations with complex traits in plants. Riedelsheimer *et al.* designed an mGWAS analysis using a set of 289 different maize inbred lines with 118 biochemical compounds and was able to identify 26 distinct metabolites that are strongly associated with single nucleotide polymorphism (SNPs) in maize, and pinpointed the key role of a chromosome 9 localized cinnamoyl-CoA reductase in improving the quality of lignocellulosic biomass [45]. In rice, a GWAS analysis using those metabolomic data obtained from 175 rice accessions successfully identified 323 associations between 143 SNPs and 89 secondary metabolites, which revealed two sorts of genetic machineries determining the natural variations in rice secondary metabolite compositions [48]. Another matrix of 840 metabolite features obtained from a worldwide collection of 524 rice accessions indicated that few loci with large effects control the levels of secondary metabolites while several loci with small effects control the natural variation of primary metabolites [49,53]. Nevertheless, although mGWAS identifies large-scale metabolite-related QTL, which maybe widely used in future in plants, several drawbacks are also inescapable at present. Firstly, limited to the present statistical algorithm, it is difficult to exactly identify the epistasis or gene-environment interaction (G × E) QTL. Secondly, limited to the precision especially in some region of the chromosome with slow decay of linkage disequilibrium, and the labor and time-consuming procedure, it is unrealistic for all of the hundreds of potential genes from one single analysis to be verified by transgenic analysis. Fortunately, the same as with other traits like seed quality, as long as the regions of interesting QTL are determined, these QTL could be further utilized for marker-assisted selection breeding without the necessarily to find out the underlying gene(s) [54].

### 2.2. Reverse Genetic Approaches for Exploring the Function of Enzyme in Certain Metabolic Pathways

Plant metabolic pathways are usually under multiple levels of regulation. Currently, our understanding of plant metabolomes results mainly from studies in a few model plants, therefore, pathways absent in those model plants are scarcely known. During the last decade, metabolomic approaches combined with reverse genetic tools (such as RNAi and gene knockout) expanded tremendously our understanding of biochemical reactions and metabolic pathways not reported in those model plants [55]. Direct measurement of the alteration in the metabolome or specific metabolic compositions of mutants can facilitate the functional annotation of the causing genes. In *Arabidopsis*, phenylalanine ammonia-lyase (PAL) is encoded by four genes involved in the phenylpropanoid pathway. Double mutant *pal1pal2* that lacks three major flavonol glycosides showed over accumulation of phenylalanine, perturbed metabolisms in other nonaromatic amino acids, as well as reduction in lignin contents [56]. Exposing the *gdh* (glutamate dehydrogenase) triple mutant to continuous darkness demonstrated that providing 2-oxoglutarate for the tricarboxylic acid cycle is the main physiological function of *NADH-GDH* (NADH-dependent glutamate dehydrogenase), and that NADH-GDH impacts remarkably on amino acid accumulation in both roots and leaves [57]. Fukushima *et al.* established a database called Metabolite Profiling Database for Knock-Out Mutants in *Arabidopsis* (MeKO) based on the metabolomic analysis on 50 *Arabidopsis* mutants, which includes images of mutants, accumulation patterns of different metabolites, as well as their statistical results [58], facilitating significantly the related studies in *Arabidopsis*. Metabolomic analyses with mutants rather than silent mutation, such as transgenic or overexpression lines, can also achieve the same outcome. In rice, constitutively overexpression of the *Arabidopsis* chloroplast *NADK* gene enhanced NADK activity, accumulated the NADP(H) pool, increased electron transport and rates of CO_2_ assimilation, and verified the critical role of NADP content in the photosynthetic electron transport rate in rice [59]. With the advancement of genome editing techniques, such as CRISPR/Cas9 [60], our understanding of a specific enzyme in plant metabolism will be significantly promoted. In addition, because genome editing is convenient and highly effective to generate multiple gene mutations simultaneously in plants [61], the interaction between two or more genes in a certain metabolic pathway can be readily explored via analyzing the metabolic profile of multiple-gene mutants.

In the future, mGWAS or mQTL analysis, combined with reverse functional genomic strategies, will more effectively uncover in depth the genetic and biochemical mechanisms governing metabolic pathways in plants.

## 3. Metabolomics and Plant Development under Normal and Stress Conditions

Successful molecular breeding largely depends on the detailed understanding of the molecular mechanisms underlying plant development obtained via systems biology approaches, including metabolomics, under normal or stress conditions. Detection of metabolic changes in different developmental stages contributes in finding characteristic metabolites (metabolic markers) for specific developmental stages. Similarly, plant metabolomics can help plant breeders to identify resistant biomarker metabolites that integrate the genetic background with the influence of the environment under stress conditions, and the selected biomarker may be used as a diagnostic metabolite for plant stress [62].

### 3.1. Spatial-Temporal Metabolic Profiling during Plant Development

The potential yield of a crop is controlled mainly by two factors, the rate of biomass accumulation and the duration of growth. Exploring dynamic metabolic changes occurring during plant growth and development may provide a new insight into the mechanisms of biomass accumulation at the metabolic level. Previous functional genomics have focused mainly on kinetics of transcripts and proteins, much less on the synchronously variable patterns of metabolites. Functional genomic analysis provides information on spatial-temporal expression patterns of genes and proteins, while metabolic profiling analysis adds informative metabolic data to functional genomic data to comprehend the whole picture of plant development. Therefore, both targeted and non-targeted metabolomic strategies have been applied in spatial-temporal metabolic profiling of developing plants. In rice, metabolomics analysis revealed substantial variation in the abundance of phenolamides, which displays developmentally controlled accumulation patterns [50]. In *Arabidopsis*, the change in the patterns of temporal-spatial distribution of the Kreb’s cycle intermediates occurs obviously in the pre-senescent leaves, and the accumulation of glucosinolates, raffinose, and galactinol occurs in the base region of leaves prior to senescence [63]. As a major part of reproductive development, seed development initiates from embryogenesis that is followed by a metabolically active period in which a massive synthesis of reserve compounds occurs in the developing seeds, whose relative proportions vary depending on the different crop seeds [33,64]. During seed development, metabolic change patterns are similar at the accumulation stage but different at the seed desiccation stage in both monocot (rice) and dicot (*Arabidopsis* and tomato), showing both conserved and divergent metabolic adaptation during plant evolution [65]. Analysis of the spatio-temporal metabolic signature of plant development is also capable to identify potential biomarkers for capturing the genetic and developmental intrinsic characteristics. Such an approach has been successfully applied to study rice tillering (branching), in which 21 metabolites captured almost 83% metabolic variation [66], and soybean developmental phase transition from vegetative to reproductive stage, in which eight flavonoid kaempferol glycosides were identified as potential growth markers [67].

Plant phenotype depends on the synthesis and accumulation of a series of metabolites in specific organs, at specific developmental stages and upon random environmental signals [68], therefore, various kinds of metabolites in the plant have organ/tissue-specific characteristics [50]. For example, sphingolipids, a class of lipids critical for male reproductive development, is significantly different between pollen and leaf tissues in *Arabidopsis* [69,70]. In young tomato seedlings, anthocyanins accumulate in hypocotyls, while several flavonols and phenolic compounds pile up in cotyledons, and some alkaloidal compounds build up in radicals/roots [68]. Since numerous biochemical components vary at different cell levels or even at subcellular levels in the plant (there are approximately 40 different cell types in plants, even a plant organ such as a leaf may include about 15 different cell types) and metabolic processes are regulated by asymmetric distribution of regulatory element (enzymes and mRNA), therefore, high resolution of spatially resolved plant metabolomic technology is increasingly required for such studies [71,72]. With the advantage of these technologies, metabolites can be traced at high spatial resolution and used to demonstrate their regulation directly [73].The application of spatially resolved metabolomic technology in plant development will be a great complement to the conventional technologies based on chromatography and mass spectrometry or NMR.

### 3.2. Metabolic Responses of Plants to Stress

Plants frequently encounter various environmental stresses during their development processes and plants have evolved a series of adaptive changes at both transcriptional and post-transcriptional levels, leading to the reconfiguration of regulatory networks to maintain homeostasis [74]. Generally, environmental stresses are classified into two types: abiotic stress and biotic stress. Abiotic stresses result from inappropriate levels of environmental factors, such as drought, flood, extreme temperature, severe radiation, metal ion stress, nutrient limitation, and oxidative stress, while biotic stresses come from pathogens and pests. Once plant receptors are stimulated by stress signals, expression of stress responsive genes is activated and subsequently specialized metabolites (especially some secondary metabolites) are biosynthesized to adapt to environmental stresses [75]. Rapid qualitative and quantitative analyses of metabolic responses of plants to environmental perturbations will help us not only to identify phenotypic response to abiotic and biotic stresses on plants and to screen for stress tolerant individuals, but also to reveal genetic and biochemical mechanisms underlying the plant’s responses to stresses, to better understand the plant plasticity for future genetic engineering of stress resistant/tolerant plants.

#### 3.2.1. Abiotic Stress

In nature, adverse environmental conditions usually consist of several different factors, and one stress is usually accompanied with or followed by another [76]. To clarify the contribution of individual stress, a controlled variable method was introduced and plants were subjected to a single primary stress factor to simplify the system [77]. Symptoms and main metabolic changes observed in single abiotic stress have been previously reviewed [78,79,80]. However, in nature, plants often encounter not only one single stress, since once a single stress occurs, it will be followed by other stresses. For example, salinity stress frequently causes osmotic stress, and flooding often leads to low-oxygen stresses [79]. Here we summarize the effects of multiple combinatorial stresses on plants, which are more similar to the natural environment. To better dissect the plant metabolic regulatory networks and their functions in the responses to complex abiotic stresses, integrated multiple-omics analysis is required [81,82,83,84]. When maize plants are subjected to water stress and salinity stress separately or concurrently, levels of six metabolites (citrate, fumarate, phenylalanine, valine, leucine, isolecuine) in leaves only change significantly under combined stresses, indicating a crosstalk effect in multiple stresses, but the potential of using those six metabolites as stress markers has not been concluded [85]. As global warming is approaching, heat and drought stresses become big challenges to sustain grain yields. A recent work on rice floral organ development provided mechanistic understandings of the responses of rice floral organs to combined stresses, in which integrative analyses on metabolomics and transcriptomic features of floral organs revealed that sugar starvation is the determinant of the failure of reproductive success under heat and drought stress in rice [86]. Heat-sensitive (Moroberekan) anther has lower levels of sucrose and myo-inositol but higher level of galactinol and raffinose, while heat-tolerant (N22) anther has lower abundances of glucose-6-P and fructose-6-P [86]. Consistent with metabolomic changes in anther, Moroberekan rice has significantly up-regulated expression of the intercellular sugar transport regulation gene Carbon Starved Anthers (CSA) [87], while N22 rice shows the enhanced expression of MST8, a sugar transporter gene, and INV4, a cell wall invertase gene [86,87]. In *Arabidopsis*, GC–MS profiling combined with transcriptomic analysis of leaves revealed a synergistic stress response of the joint treatment of darkness and high temperature, which is attenuated by low temperature. Because protein degradation occurs rapidly, amino acid catabolism comes to be the main cellular energy supply in the absence of photosynthesis, as evidenced by the conditional connections between amino acid metabolism and the Kreb’s cycle [82]. The combined cold and dehydration stresses in rice cause the up-regulation of carbohydrate metabolism associated genes, which is consistent with the buildup of glucose, fructose, and sucrose in the aerial parts of the plant [83]. Several sugars such as sucrose, raffinose, maltose, and glucose frequently accumulate in plant cells suffering combinatory stresses, perhaps protecting plants via osmotic adjustment from oxidative damage that usually follows most stress conditions [88,89]. Combined stresses normally result in a more extreme condition than that of each individual stress alone, and therefore has profound effects on central metabolisms such as sugars and their phosphates and sulfur-containing compounds [82,88,89]. Interestingly, the combined elevated CO_2_ and salinity stress exerts a milder effect on the metabolic physiology of the plants than that of salinity stress alone [84], indicating that there is a complicated crosstalk between different stresses, which merits further investigations. In addition, a recent study revealed that secondary metabolism is also involved in the plant’s tolerance to the combinatorial drought and salinity stresses, in which the tolerant Tibetan wild barley (XZ25 and XZ16) displays transcriptomic alterations in the levels of secondary metabolism pathway genes and lower DNA damage, as compared with control barley cv CM72, together with an increase of flavonoids and phenols [90].

#### 3.2.2. Biotic Stress

To combat attacks from pathogens and pests, plants use complex chemical machinery as a major defense. Similar to distinctive responses to diverse abiotic stresses, metabolic responses of plants to biotic stresses depend also highly on tissues, species, and plant-pathogen or pest interactions. Consequently, the identified compounds from biotic stressed plants help in searching for novel defense compounds, and meanwhile serve as important plant defensive state markers [91]. Increasing numbers of metabolites have been identified and regarded as biotic stress tolerant or sensitive metabolic biomarkers in diverse plant species. For example, 16 fatty acids (such as unsaturated linoleic acid) together with two amino acids (glutamine and phenylalanine) were identified as the major components of the resistance features of gall midge resistant rice varieties [92]. When subjected to BLB (bacterial leaf blight) caused by *Xanthomonas oryzae* pv. *oryzae* (*Xoo*), sensitive and tolerant rice cultivars display contrasting changes in several specific metabolites, such as acetophenone, xanthophylls, alkaloids, carbohydrates, and lipids [93]. The agent of rice blast disease, *Magnaporthe grisea*, another devastating pathogen of rice, can also infect other important crops, including wheat, barley, and purple false brome grass [94]. Metabolomic analysis revealed identical changes in metabolic patterns in barley, rice, and purple false brome grass, in which malate, polyamines, quinate, and non-polymerized lignin precursors accumulate during infection by *M. oryzae* [95]. The accumulation of phenylpropanoid and phenolic compounds is also reported in response to *F. graminearum* in wheat [96]. Phenylpropanoids, the precursors of lignin, constitute an important component of plant stress defense mechanism, which modulate cell wall composition and stiffness in root. The thickened cell wall may help to defend against pathogen infection in the plant. In the future, metabolomics will focus on a better understanding of the chemical machinery operating in plants responding to both abiotic and biotic stresses and the improvement of plant resistance, thus reducing crop yield lost under stress conditions.

## 4. Application of Plant Metabolomics in Plant Society Other than Basic Research

### 4.1. Safety Assessment of Genetically Modified (GM) Crops

The application of genetic engineering to produce genetically modified (GM) crops is considered one of the most developed leading agro-biotechnologies. Though GM crops have been proven to have huge economic potential considering their effects on value-added traits such as tolerance to herbicide, resistance to insects, faster or delayed ripening, high levels of antioxidants and other nutrients, authorization and commercialization of GM crops have always been controversial among both the scientific community and the public sector over their potential risks to the environment and human health [97]. Therefore, risk assessments of GM plants and derived products are very strict in many countries and regions including the European Union. Metabolomics provides an additional dimension to GM crop analysis, allowing the detection of both intended and unintended effects that might take place in GM crops because of genetic modification at the metabolic level. This facilitates greatly the substantial equivalence evaluation of GM crops. Case studies comparing metabolic changes between GM crops and their non-transgenic counterparts have covered almost all important agricultural crops, such as rice, maize, soybean, pea, wheat, potato, tomato, barely, and so on, which have confirmed detectable alterations of their metabolites due to transgenic modifications [97,98]. However, results also show that other factors such as environmental conditions usually exert greater influences on metabolic compositions than genetic modification [99]. Therefore, in this case, metabolomics studies comparing GM crops with their non-GM counterpart lines are often combined with parallel studies using different culture conditions, in different geographical locations, and over multiple years, to corroborate the authentic effect of genetic modification [99]. Recently, natural variation has been taken into consideration for the substantial equivalent assessment of GM crops. If the variations between a GM crop and its non-GM counterpart parent lines fall in the range of natural variation, then it is considered to be safe at metabolic level [100]. However, for detecting unintended effects caused by genetic modification in GM crops, non-targeted metabolomics seemed to be more powerful than targeted metabolomics [101]. Nevertheless, the applications of non-targeted metabolomics in GM crops have not been validated and approved yet within the global regulatory framework for GM food safety assessment [97]. In the future, to facilitate the molecular characterization of GM crops at the metabolomic level, multiple metabolomic platforms to detect as many as possible metabolites in a sensitive and robust manner [102], and the exploration of the metabolomic variation in more and more crops rather than just rice [10] and maize [12], such as soybean [103,104] are needed.

### 4.2. Metabolomics and Crop Improvement

Crop breeding depends largely on phenotypic selection in plots or genomic selection by genetic markers. This is hindered by great hurdles, for example, marker effects for selecting complex traits vary frequently among populations [105]. Metabolomics combined with other omics will allows us to solve key issues of agronomic performance that remained unsettled previously. Efforts can be directed to crop plants that have detailed information on performance in large-scale environments [106]. The information resulting from mQTL and mGWAS allows us to analyze the nature of quantitative traits of interest. Plant metabolomic technology can provide information not only on the numbers of identified metabolites but also their correlations with each other and with agronomic important traits, thus it could lead to the development of more rational models to link specific metabolite or pathway with yield or quality associated traits. Even more promising is the possibility of studying the relationship between metabolite variations and the resulting phenotypes [107]. Notably, the ongoing efforts elucidating the metabolic responses to various stresses imply that metabolomics-assisted breeding could also be useful in obtaining crops more resistant to stresses [108]. The important role of metabolomics in crop improvement will become increasingly evident in the future.

### 4.3. Plant Improvement by Metabolic Engineering

Since plants are capable of designing and producing multifarious chemical compounds that serve mankind as foods and medicines, effective engineering of metabolic pathways in plants associated with modern biotechnology will bring more benefits to human beings [109]. As a successful example, golden rice that accumulates higher levels of vitamin A proves that the nutrient content of a crop plant can be improved by metabolic engineering [110]. However, due to the limitation of current knowledge about metabolic control, it is quite challenging to rationally engineer complicated metabolic networks. Recent technological advances in plant metabolomics and other “omics” offer golden opportunities to dissect the remarkable complicacy of the plant biochemical capacity and facilitate a better investigation into plant metabolic systems to increase the potential of practical applications through precise metabolic engineering [111]. Based on knowledge of sugar biosynthesis and accumulation pathways, yields of endogenous sugars, such as higher-value sugars and simple sugar derivatives, have been successfully increased via plant metabolic engineering [112]. Knowledge-based metabolic engineering strategies, generating large datasets and rational models of metabolic pathways via large scale gathering and mining of various omics data, will continuously help to refine the input and output of engineering plants [113].

## 5. Conclusions and Future Perspectives

With the growing interest in the use of metabolomic technologies for a wide range of biological targets, plant metabolomics have dramatically improved in recent years. The combination of the capabilities of available analytical platforms for the analyses of complex samples, together with the integration of metabolomics with other “omics” and functional genetics, is able to provide novel insights into genetic and biochemical aspects of cellular function and metabolic network regulation [114]. Plant metabolomics, alone or combined with functional genomics, has been applied in many fields. Even though it has some limitations currently, it is no doubt an important tool that is revolutionizing plant biology and crop breeding.

**Figure 1:**
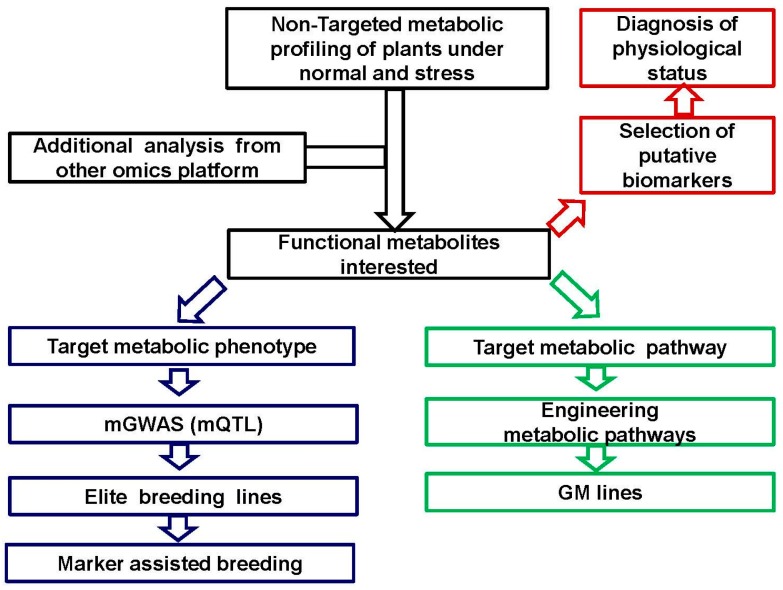
The schematic presentation of plant metabolomics and its application in plant improvement.

The full elucidation of biochemical and genetic mechanisms underlying plant developmental and stress responsive biology depends largely on the comprehensive investigations using systematic omics techniques, which is the foundation for the application of metabolomics in plant science. Among them metabolomics is of particular importance, because the metabolites are more relevant to the plant phenotype (both physiological and pathological phenotypes) as compared with DNAs, RNAs or proteins [115]. Therefore, future studies in this area will focus on both directions: one is the improvement of the metabolomic platform to facilitate the accurate and effective identification and quantification of as many as possible metabolites (mainly secondary metabolites), the precise interpretation of generated data, and the rapid integration with other omics platforms; the other is the comprehensive investigation into molecular and biochemical mechanisms of metabolic variations in plants (mainly crops) using both non-targeted and targeted approaches, to expand and enrich the understanding of plant metabolism in growth and development under both normal and stressed conditions, and the application of metabolomics to plant breeding (Figure 1) for better crop yield and quality.

## Figures and Tables

**Table 1:** Summary of mQTL (metabolite quantitative trait loci) and mGWAS (metabolome-based genome-wide association study) studies in plant.

Species	Tissue	Population Type	Method	Metabolic Traits	Ref.
mQTL study					
*Arabidopsis*	Harvested seed	Recombinant inbred lines	HPLC	Tocopherol	[27]
*Arabidopsis*	Leaf	Recombinant inbred lines	GC–TOF-MS	Metabolome	[28]
*Arabidopsis*	Seed	Recombinant inbred lines	LC–MS	Flavinoids	[29]
*Arabidopsis*	Seedling	Recombinant inbred lines Introgression lines	GC–TOF-MS	Metabolome	[21]
*Brassica napus*	Leaf Seed	Doubled haploid lines	HPLC	Glucosinolates	[25]
Maize	Leaf	Recombinant inbred lines Natural accessions	GC–TOF-MS	Primary Metabolites	[30]
Rice	Seed	Chromosomal segment substitution lines	LC-Q-TOF-MS	Metabolome	[24]
Rice	Seed	F2, F2-derived lines	GC–MS	Lipids	[31]
Rice	Flag leaf Germinating seed	Recombinant inbred lines	LC–EI–MS	Metabolome	[32]
Tomato	Fruit	Introgression lines	GC–MS	Metabolome	[22]
Tomato	Fruit	Introgression lines	GC–MS	Metabolome	[33]
Tomato	Fruit	Introgression lines	GC–MS, LC–MS	Metabolome	[34]
Tomato	Fruit	Introgression lines	GC–MS	Primary Metabolites	[35]
Tomato	Fruit	Introgression lines	UPLC	Secondary Metabolites	[36]
Wheat	Flag leaf	Doubled haploid lines	LC–ESI–MS	Metabolome	[23]
Wheat	Flag leaf	Doubled haploid lines	GC–MS	Metabolome	[37]
mGWAS study					
*Arabidopsis*	Seed	Natural accessions	LC–MS	Branched-chain amino acids	[38]
*Arabidopsis*	Leaf, Seedling	Natural accessions	LC–MS	Glucosinolates	[39]
*Arabidopsis*	Leaf	Natural accessions	GC–TOF-MS	Metabolome	[40]
Maize	Kernel	Natural accessions	UPLC–MS	Metabolome	[41]
Maize	Grain	Natural accessions	HPLC	Carotenoid	[42]
Maize	Grain	Natural accessions	HPLC	Tocochromanol	[43]
Maize	Leaf	Natural accessions	GC–MS	Metabolome	[44]
Maize	Leaf	Natural accessions	GC–MS	Metabolome	[45]
Maize	Kernel	Natural accessions	LC–MS	Metabolome	[46]
Potato	Tuber	Natural accessions	GC–MS	Primary Metabolites	[47]
Rice	Leaf	Natural accessions	LC–QTOF-MS	Secondary Metabolites	[48]
Rice	Leaf	Natural accessions	LC–MS	Metabolome	[49]
Rice	Leaf	Natural accessions	LC–MS	Phenolamides	[50]
Tomato	Fruit	Natural accessions	GC–MS	Metabolome	[51]
